# Developing a Food Exposure and Urine Sampling Strategy for Dietary Exposure Biomarker Validation in Free‐Living Individuals

**DOI:** 10.1002/mnfr.201900062

**Published:** 2019-06-17

**Authors:** Amanda J. Lloyd, Naomi D. Willis, Thomas Wilson, Hassan Zubair, Long Xie, Edward Chambers, Isabel Garcia‐Perez, Kathleen Tailliart, Manfred Beckmann, John C. Mathers, John Draper

**Affiliations:** ^1^ Institute of Biological Environmental and Rural Sciences Aberystwyth University Aberystwyth SY23 3DA UK; ^2^ Human Nutrition Research Centre Institute of Cellular Medicine Newcastle University Newcastle‐upon‐Tyne NE2 4HH UK; ^3^ Nutrition and Dietetic Research Group Division of Diabetes Endocrinology and Metabolism Department of Medicine Hammersmith Hospital Campus Imperial College London W12 0NN UK

**Keywords:** biomarkers, dietary exposure, free‐living, metabolomics, urine sampling

## Abstract

**Scope:**

Dietary choices modulate the risk of chronic diseases and improving diet is a central component of public health strategies. Food‐derived metabolites present in urine could provide objective biomarkers of dietary exposure. To assist biomarker validation, this work aims to develop a food intervention strategy mimicking a typical annual diet over a short period of time and assesses urine sampling protocols potentially suitable for future deployment of biomarker technology in free‐living populations.

**Methods and results:**

Six different menu plans comprehensively represent a typical UK annual diet that is split into two dietary experimental periods. Free‐living adult participants (*n* = 15 and *n* = 36, respectively) are provided with all their food, as a series of menu plans, over a period of three consecutive days. Multiple spot urine samples are collected and stored at home.

**Conclusion:**

A successful food exposure strategy is established following a conventional UK eating pattern, which is suitable for biomarker validation in free‐living individuals. The urine sampling procedure is acceptable for volunteers and delivered samples suitable for biomarker quantification. The study design provides scope for validation of existing biomarker candidates and potentially for discovery of new biomarker leads, and should help inform the future deployment of biomarker technology for habitual dietary exposure measurement.

## Graphical abstract

**Food‐derived metabolites** present in urine could provide objective biomarkers of dietary exposure. A food intervention strategy mimicking a typical diet in free‐living individuals is developed and spot urine sampling protocols are assessed. This study provides scope for validating existing biomarker candidates and discovering new biomarker leads, and should help in deploying biomarker technology for dietary exposure measurement in the future.

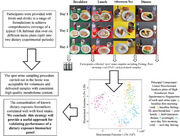

## 1 Introduction

An unbalanced diet and physical inactivity are important risk factors in the development of chronic diseases including cardiovascular diseases (CVD), type 2 diabetes, many cancers, dementia, and musculoskeletal diseases and drive the increase in obesity prevalence. Consequently, improving dietary choices is a cornerstone of national and international strategies for reducing chronic disease burden.^1^, ^2^ A key factor in effective implementation of public health strategies is the need for validated population screening methods with which to determine the effectiveness of interventions to change individual's dietary intake and the effectiveness of industry to improve the composition of foods, meals, and diets. For a clear understanding of the relationship between food exposure and health status or disease risk there is a need for accurate monitoring of diet which can be recorded using self‐reported measures such as Food Frequency Questionnaires (FFQs), 24‐h recall and diet diaries/records. However, these methods are subjective, misreporting is common and substantial and is exacerbated in those who are overweight or obese.^3^, ^4^ Recent research has demonstrated that metabolites derived from individual foods present in urine samples provide potential biomarkers of dietary exposure, and including such measurements could overcome some of the limitations of traditional dietary assessment methodologies by providing additional objective estimates of food exposure.^5^ A range of factors have been proposed that need to be considered for the validation and deployment of dietary exposure biomarkers.^6^ Fully validated dietary exposure biomarkers which are robust and reproducible and have been tested in both free‐living and controlled food studies, within different food matrices are limited to a relatively small number of specific foods and food components.^7^, ^8^, ^9^ The use of multi‐metabolite biomarker panels may provide more reliable estimation of dietary exposure than a single‐biomarker approach (reviewed by ref. 10). For dietary biomarkers to have any significant utility, it is essential that their coverage is as comprehensive as possible. Nationally representative estimates of intakes of foods by the UK population are provided by the UK National Diet and Nutrition Survey (NDNS),^11^ and can be explored to indicate where dietary exposure biomarker discovery might be feasible and relevant.

Dietary exposure biomarkers ideally need to be suitable for use in large‐scale surveys and epidemiological studies. Any urine sampling procedure would need to be acceptable for volunteers to provide repetitive samples and require minimal researcher time but deliver samples with high‐quality information content. To date there are no studies focusing on dietary exposure biomarker validation that have investigated specifically natural micturition behavior and acceptability of urine sampling methods in free‐living populations as a prelude to biomarker technology deployment under real world conditions. A recent review of nutrimetabolomics suggested that first morning void, post‐prandial spot, or 24‐h urines can be collected, depending on the purpose of the study.^12^ The “gold standard” method of requesting participants to collect all urine over a 24‐h period^13^ provides a substrate for accurate quantitation of food intake during a single day using biomarkers, but imposes a significant burden on participants by impacting on normal daily activities, as well as being costly and logistically difficult to handle large volumes of urines by both participants and researchers. Recently it has been shown that spot fasting sample collections, which are low burden for participants, can adequately discriminate exposure class for several dietary components, and could possibly substitute for 24‐h urine samples for biomarker discovery and habitual dietary exposure measurements.^14^ In acute food intervention dietary biomarker discovery experiments, we have shown that 3‐h postprandial urines provided strong classification models^15^ but because urine composition was relatively stable over a period of 2–4 h after eating a meal, there may be a flexible time window for urine collection.^16^ The MAIN (Metabolomics at Aberystwyth, Imperial and Newcastle) study aimed to identify foods and food groups for which future biomarker detection and validation is feasible and used this information to develop two complex 3‐day menu plans, in the context of conventional UK eating patterns. Additionally we aimed to design and implement urine sampling protocols and procedures which could be applied in future large‐scale epidemiological studies and public health surveys.

## 2 Subjects and Methods

### 2.1 Ethical Approval

A favorable ethical opinion was obtained following Proportionate Review by the East Midlands ‐ Nottingham 1 National Research Ethics Committee (14/EM/0040). Caldicott approval for storage of data and data protection was granted by Newcastle‐upon‐Tyne Hospitals NHS Foundation Trust [6896(3109)]. The trial was adopted into the UK Clinical Research Network (CRN) Portfolio (16037) and is registered with International Standard Randomized Controlled Trials Number (ISRCTN), 88921234. All participants gave written informed consent, and the study was carried out in accordance with the Declaration of Helsinki. International Standard Randomized Controlled Trials Number (ISRCTN), 88921234.

### 2.2 Study Design and Urine Sampling

Previously, participant handling protocols were developed in which acute exposure to specific foods of high public health importance was investigated under carefully supervised conditions.^16^, ^17^, ^18^ Similar protocols were used in the present study but they were adapted for use with free‐living participants. Some behavioral restrictions were imposed on volunteers on the day preceding the test day. Participants were asked to restrict physical activity and to avoid alcohol consumption and to restrict polyphenol intake.

Foods/food groups were selected using information on consumption frequencies, food groupings, and eating habits from the UK NDNS years 1–3,^11^ together with Public Health England policy advice from The Eatwell Plate^19^ which has now been revised to The Eatwell Guide^20^ (as described in the Results section in **Table** 1 and Supporting Information 1). Standard portion sizes were used based on the UK Food Standards Agency^21^ or manufacturers’ suggestions. Six different menu plans were designed using a design strategy described in Supporting Information 2, that were investigated in two dietary experimental periods in which healthy free‐living adult participants (experimental period 1, *n* = 15 where 53 female, non‐smokers, age = 22–63; experimental period 2, *n* = 36 where 58% female, non‐smokers, age = 19–77 years) were provided with all their food for the three consecutive days (**Table** 2 shows an example menu plan and Supporting Information 3 shows the full menu plans). A sample size of 15 participants was aimed for in the first experimental period 1 and 30 participants in the second experimental period to allow for a 20% drop out. This sample size was determined by the feasibility of recruitment. The menu plans were designed to provide 4–5 key targeted foods each day (Supporting Information 1 and 2). Energy and macronutrient contents of the meals and daily menus (Supporting Information 4) were calculated directly from food packaging and by using the USDA National Nutrient database (for perishable foods sold without packaging).^22^

**Table 1:** Section of the database compiled for the selection of foods/food groups for biomarker development Values in brackets are the percentage of the population whom recorded consumption of the food item ≥ 1

Food type	Disaggregated food groups in UK national diet and nutrition survey (NDNS)	Top three food choices for each NDNS food group
Fruit	Fresh and canned fruit (92%)	Bananas, raw
Fruit		Eating apples, raw
Fruit		White grapes, raw
Fruit	Fruit juice (87%)	Orange juice, UHT
Fruit		Orange juice, pasteurized
Fruit		Robinsons juice
Fruit	Dried fruit (45%)	Raisins
Fruit		Sultanas
Fruit		Stuffing (filling)
Vegetable	Yellow/red & dark green leafy vegetables (93%)	Carrots, boiled
Vegetable		Peppers, red, boiled
Vegetable		Carrots, uncooked
Vegetable	Tomato (91%)	Tomatoes, raw
Vegetable		Baked beans
Vegetable		Canned tomatoes
Vegetable	Tomato puree (83%)	Baked beans
Vegetable		Tomato ketchup
Vegetable		Cheese and tomato pizza
Vegetable	Brassicaceae (64%)	Broccoli spears, boiled
Vegetable		Cauliflower, boiled
Vegetable		Mixed leaf salad
Vegetable	All other vegetables (97%)	Onions, boiled
Vegetable		Cucumber, raw
Vegetable		Lettuce, raw

**Table 2:** The menu plan for experimental day 1

Meal	Time	Menu
Breakfast	08:00–10:00	Instant coffee
		1 slice of sourdough rye bread toasted with butter
		Frosties with Semi skimmed UHT milk
		Banana
Lunch	12:00–14:00	Instant coffee
		Tuna chunks as a salad with lettuce, onion and sweetcorn
		2 slices of sourdough rye bread with butter
		Banana
Afternoon tea	16:00–16:30	Instant coffee
		Banana loaf
Dinner	18:00–20:00	Grilled salmon steak with broccoli and chips
		Coca cola
		Red wine
		Almonds
		Bottled water, throughout day and at each meal time

**Figure 1:**
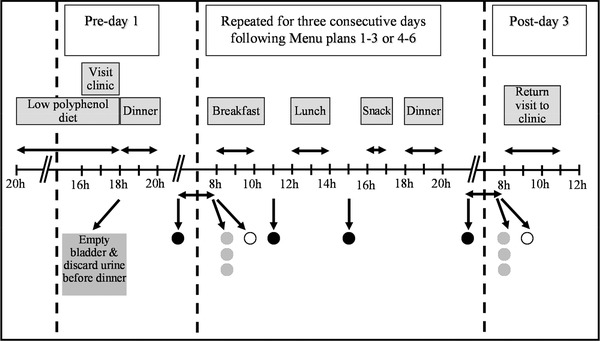
MAIN study experimental period. Participants were asked to consume a low polyphenol meal (provided or of the participants own choice) in the evening prior to starting each 3‐day menu plan (pre‐day). All foods and drinks for four meals each day for three consecutive days were provided. These foods/drinks were prepared, cooked, and consumed by the participant at home. Participants were asked to collect all urines as separate “spot urines” in specific time‐windows including (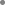) first morning void, FMV; (○), fasting; (●) and post‐prandial urines (post‐breakfast, post‐lunch, and post‐dinner) for the duration of the experimental period.

The first day of the first experimental period 1 was based largely on foods/food groups for which putative dietary exposure biomarkers were already available. The remaining five menu plans aimed to deliver foods for new biomarker discovery, and to consider the impact of the major sources of likely variance in biomarker monitoring and discovery, including impact of complex and mixed meals, different food formulations, processing, and cooking methods as well as the dynamics of putative biomarker retention in the body. Menus plans were designed in the context of conventional UK eating patterns viz. breakfast, lunch, afternoon snack, and dinner (**Figure** 1). The first three menu plans (in the first experimental period) were repeated by the participants twice in the same order but a week apart. The remaining three menu plans (in the second experimental period) were repeated by the participants three times in a randomized 3×3 Latin square design. Participants came to the Clinical Ageing Research Unit (CARU), Newcastle University on the day before the experimental period to collect foods in pre‐determined food portions or “ready meals” (as appropriate), and cooking instructions and to be briefed. These foods/drinks were prepared, cooked, and consumed by participants at home. Participants were requested not to consume any foods or drinks, including alcohol, other than those provided by the research team and to eat the meals within the stated times each day. Water, however, was allowed ad libitum and participants were encouraged to remain hydrated throughout. Participants were requested to record the time that they finished each meal and how much of each food/drink item they ate and at what time. If some food was uneaten, the participant recorded eating 75%, 50%, 25%, or 0%, as appropriate. Participants recorded if they ate or drank any additional items or prepared any of the meals differently from that instructed. Further study details will be described in a future publication.

Urine sampling methods were implemented based on the previous studies.^16^, ^18^ Participants were asked to collect urine samples after their evening meal (post‐dinner), all urines overnight including the first morning void (FMV), fasting, and post‐breakfast and post‐lunch urine samples up to the day after the completion of the menu plans (Figure 1). Participants collected urine in a calibrated plastic jug and recorded the date, time, and total volume of collection. A 20‐mL aliquot from each urination was retained and the rest discarded. If not at home, participants kept urine samples in a cool bag, otherwise they stored them in a refrigerator before returning them to the research team at the end of each experimental week, where they were further stored at −80 °C until analysis. An overnight urine sample was made by pooling aliquots of each urine proportional to the volume of urine collected after dinner, overnight and including the FMV and stored at −80 °C.

### 2.3 Urine Sample Preparation and Adjustment

All urine samples were normalized by refractive index prior to analysis to ensure all MS measurements were made within a similar dynamic range. Samples were defrosted overnight in a 4 °C fridge. Once defrosted, samples were centrifuged (600 × *g* for 5 min at 4 °C), placed on ice and aliquots of thawed urine (1000 µL) was transferred into labelled 2‐mL Eppendorf tubes. The remaining samples were returned to a −20 °C freezer. An OPTI Hand Held Refractometer (Bellingham Stanley Brix 54 Model) was calibrated with de‐ionized water (dH_2_O) and dried with paper tissue according to the manufacturer's instruction. Following this 220 µL of sample was transferred onto the refractometer dish, the specific gravity (SG) value was recorded in triplicate and temperature was noted. The refractometer was rinsed with dH_2_O between samples and dried with tissue. Average SG values were calculated. Based on these figures, aliquots of the required amounts of urine from centrifuged 2‐mL Eppendorf tubes and dH2O were transferred into new tubes for extraction.

### 2.4 Flow Infusion‐High Resolution Fingerprinting (FIE‐HRMS)

All urine samples were randomized according to meal day and post‐prandial time point to minimize batch effects and were analyzed using high resolution (HR) flow infusion electrospray (FIE) ionization mass spectrometry (MS). 20 µL of extracted sample was transferred to a glass HPLC vial containing a 200 µL flat bottom micro insert (Chromacol) and diluted with 80 µL of H_2_O:MeOH (3:7) directly in the vial. Mass spectra were acquired on an Exactive Orbitrap (ThermoFinnigan, San Jose CA) mass spectrometer coupled to an Accela (ThermoFinnigan, San Jose CA) ultra‐performance liquid chromatography system. 20 µL of sample was injected and delivered to the electrospray source via a flow solvent (mobile phase) of pre‐mixed HPLC grade MeOH (Fisher Scientific) and ultra‐pure H_2_O (18.2 Ω) at a ratio of 7:3. The flow rate was 200 µL min^−1^ for the first 1.5 min, and 600 µL min^−1^ for the remainder of the protocol, total time 3.0 min.

Positive and negative ionization modes were acquired simultaneously. For each ionization mode, one scan event was used to acquire all mass spectra, 55.000–1000.000 *m/z* and 63.000–1000.00 *m/z* for positive and negative mode respectively. The scan rate was 1.0 Hz. Mass resolution was 100 000, with automatic gain control 5×10^5^ and maximum injection time 250 ms, for both ionization modes. Following data acquisition, raw profile data (.raw; ThermoFinnigan) were converted to the .mzML open file format and centroided^23^ using msconvert (TransProteomicPipeline).^24^ All further processing of mzML files was performed using the R Statistical Programming Language using the R package binneR (Version 1.1.0).^25^

Dimensionality reduction of the acquired mass spectra was performed by taking each *m/z* value from scans about the apex of the infusion profile and binning the *m/z* and intensity values at 0.01 amu intervals, allowing direct comparison of urine fingerprints, prior to signal annotation. The result was an *n x p* matrix, where *n* is the sample and *p* is the *m/z* feature and cells are the respective average intensity values. The resulting matrices consisted of 4552 features and 4857 features for positive and negative ionization modes respectively. The total number of observations for each ionization mode was 633.

### 2.5 Multivariate Modelling and Classification

Principal Component Analysis (PCA) was followed by PC‐Linear Discriminant Analysis (PC‐LDA). PCA was performed using the *prcomp* function in R.^25^ Intra‐batch variance was removed adjusting intensity values by the mean value of the respective analytical block (where each block contains an equal representation of the total biological variance). Prior to statistical analysis, data was normalized to the total ion count (TIC) of the sample. For multivariate analysis (PCA and PC‐LDA) all samples and features were used. Plots of the first two PC‐Discriminant Functions (PC‐DFs) allowed visualization of the goodness of class separation as quantified by *T*_w_ values (Eigenvalues). Supervised Random Forest (RF) classification was implemented using the randomForest package in R.^25^ For all Random Forest models, the number of trees (*ntree)* used was 1000 and the number of variables considered at each internal node (mtry) was the square root of the total number of variables. Accuracy, margins of classification, and area under the ROC (Receiver Operator Characteristic) curve (AUC) were used to evaluate the performance of classification models, as previously described.^26^ Models were deemed adequate overall if RF margins > 0.2 and AUC values > 0.8, as it has been implemented previously.^26^, ^27^

### 2.6 Quantification Using Ultra High Performance Liquid Chromatography (UHPLC)

Biomarkers were quantified in urine samples by a procedure based on Multiple Reaction Monitoring.^28^ Analyses were performed on a TSQ Quantum Ultra triple quadrupole (QQQ) mass spectrometer (Thermo Scientific), equipped with a heated electro‐spray ionization (HESI) source and coupled to an Accela UHPLC system (Thermo Scientific). Columns used for separation of individual biomarkers together with retention times are shown in Supporting Information 5. For HILIC (Hydrophilic Interaction Liquid Chromatography) analysis, chromatographic separation was performed on a ZIC‐pHILIC (polymeric 5 µm, 150 × 4.6 mm) column (Merck). The mobile phase consisted of A) 10 mm ammonium acetate in water: acetonitrile (95:5) and B) 10 mm ammonium acetate in water: acetonitrile (5:95). The gradient program used was as follows: 0 min, 95% B (400 µL min^−1^); 15 min, 20% B (400 µL min^−1^); 15.01 min, 20% B (500 µL min^−1^); 20 min, 20% B (500 µL min^−1^); 20.01 min, 95% B (500 µL min^−1^); 25 min, 95% B (500 µL min^−1^). The HPLC was carried out in low pressure (≈0–7000 psi) operating mode with 0 and 650 bar as minimum and maximum pressures, respectively. For reverse phase (RP) analysis, chromatographic separation was performed on Hypersil Gold (1.9 µm, 200×2.1 mm^2^) (Thermo Scientific). The mobile phase consisted of A) 0.1% formic acid in H_2_O and B) 0.1% formic acid in MeOH. The gradient program used was as follows: 0 min, 0% B; 0.5 min, 0% B; 5 min, 60% B; 11 min, 100% B; 13 min, 100% B; 13.01 min, 0% B; 19 min, 0% B. For RP analysis, the flow rate was maintained at 400 µL min^−1^. The UHPLC was carried out in high pressure (≈7000–15 000 psi) operating mode with 0 and 1000 bar as minimum and maximum pressures, respectively. For both chromatographic analyses, column oven and autosampler tray were maintained at 60 and 14 °C, respectively. To ensure consistent sample delivery, 20 µL were injected using a 20 µL loop and partial loop injection mode. After each injection, syringe and injector were cleaned using a 10% HPLC grade MeOH solution in ultra‐pure water (1 mL flush volume) to avoid sample carryover. Spectra were collected at a scan speed of 0.010 and 0.003 s for HILIC and RP analysis, respectively. A scan width of 0.010 u, and peak width (Q1, Q3) of 0.7 FWHM were used for both HILIC and RP analyses. Mass spectra were acquired in multiple reaction monitoring (MRM) mode, in positive and negative ionization mode simultaneously using optimized values of shimmer offset, collision energy, and tube lens for each MRM transition (see Supporting Information 5 for transitions). Limit‐of‐detection (LOD) and limit‐of‐quantification (LOQ) are shown in Supporting Information 5. Absolute concentrations were calculated using a nine‐point calibration curve (0.006561 to 100 µg mL^−1^) for each biomarker). Mean concentration of biomarkers for selected dietary components between the different menus plans were tested for significance using the paired *t*‐test significance values. Xcalibur (V3.0.63, Thermo Fisher Scientific Inc.) was used for peak integration, calibration, and quantification. A squared fit of log 10‐transformed values accommodated best the wide concentration range for biomarkers in high and low consumers, without compromising accuracy and normal distribution requirements for regression analysis.

## 3 Results

### 3.1 Selection of Foods/Food Groups Used in Menu Design for Urine Biomarker Discovery and Validation

Current approaches used for biomarker discovery generally involve metabolomic comparisons of samples derived from either controlled dietary interventions or cross‐sectional studies. Supporting Information 6 summarizes the major characteristics of typical dietary exposure biomarker discovery strategies. In most instances significant validation challenges remain, particularly in relation to biomarker specificity and sensitivity in the context of normal population eating behavior. Here we used quantitative information on individual‐level food intake in the UK^11^ and UK healthy eating advice^19^ (Table 1 and Supporting Information 1) to select candidate foods/food groups for incorporation into two complex 3‐day experimental periods in the context of conventional UK eating patterns. Importantly this included information on cooking/preparation methods of commonly consumed foods which we used to test biomarker generalizability by using different formulations/cooking/processing methods for these foods. As an example, the fruit and vegetable subsection of the database is shown in Table 1. The menu design strategy, which is further described in Supporting Information 2, revealed target foods and food groups for incorporation into the menu design to facilitate future biomarker discovery for these food groups (Table 2 and Supporting Information 3). We aimed to incorporate the most commonly consumed foods in each category using the same cooking/processing methods as reported in the NDNS data (Table 2 as an example of 1 menu plan and Supporting Information 3).

### 3.2 Validation of Acceptability of Menu Design

Participants recorded how much of each food/drink item they consumed and this compliance information is summarized in Supporting Information 7. Overall, there was a high compliance (>80%) for each food item in all menus. The lowest mean compliance was for instant coffee (80%), followed by soya milk drink and apple pie (both 82%).

### 3.3 Developing a Strategy for Collection of Informative Spot Urine Samples at Home

**Figure 2:**
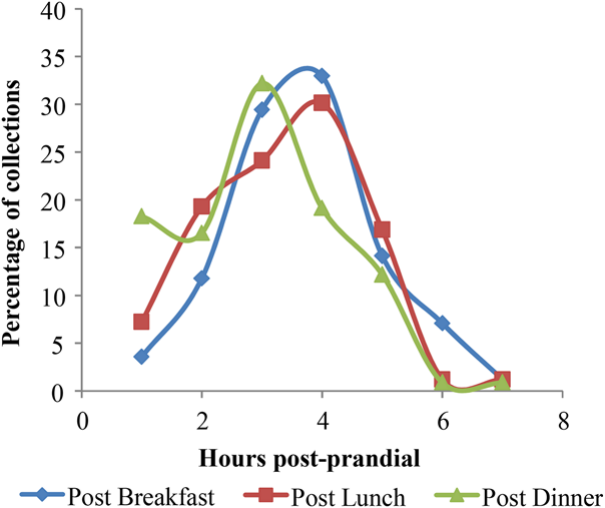
Timings of post‐prandial urine sample collections for 15 participants following menu plans 1–3 over two weeks (expressed as a percent of collections at each time‐point).

We calculated the compliance of urine sample collections for 15 participants following menus 1–3 over two weeks. The most successful urine type collected was FMV and the post‐dinner spot sample (both at 99% compliance). Post‐prandial samples had a compliance of between 92–99% (Post‐breakfast, 93%, Post‐lunch 92% and post‐dinner, 99%). The fasting sample was the least successfully collected sample (86%). Most urine samples were collected between 2 and 4 h after each main meal (breakfast, lunch, and dinner; **Figure** 2). On some occasions, participants collected multiple post‐breakfast and post‐lunch urine samples (8% and 16%, respectively), when only a single sample was requested. In addition, on 75% of occasions, participants collected multiple post‐dinner samples as requested in the protocol. The overnight micturition patterns (data shown in Supporting Information 8) were even more variable between individuals. The most common number of urines produced after the evening meal was 3 or 4 (28% and 32% respectively) reaching a maximum of 6 in 3% of study group.

### 3.4 Metabolome Fingerprint Analysis to Assess Compositional Similarities between Urine Types

**Figure 3:**
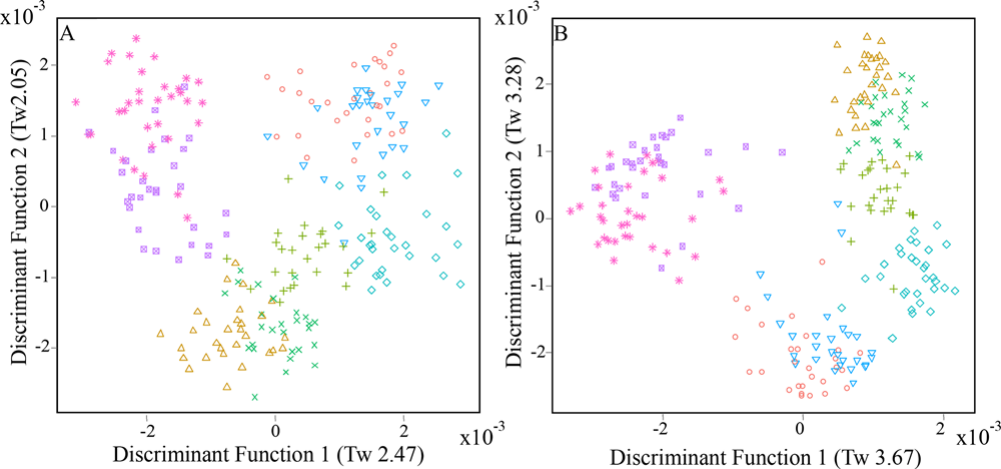
Principal component‐linear discriminant analysis (PC‐LDA) scores plots of flow infusion electrospray ionization‐high resolution mass spectrometry (FIE‐HRMS) fingerprints of each urine sample collected on the experimental day and the FMV and fasting urines collected the next morning for 15 participants following menu plan 1 A) positive ionization mode; B) negative ionization mode. PC‐Discriminant Function (PC‐DF) Eigenvalues (*T*_w_ values) are given in brackets; x, baseline first morning void; ∆, baseline fasting; 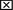, post‐breakfast; *, post‐lunch; ○, bed‐time/post‐dinner; ∇,overnight◇, next day first morning void; +, next day fasting.

Metabolome fingerprints were generated by non‐targeted FIE‐HRMS to determine the chemical compositional similarity between the behavioral phase urine types using supervised, multivariate classification tools including Random Forest (RF) and Principal Component Linear Discriminant Analysis (PC‐LDA). Where multiple post‐prandial spot urine samples were collected, we selected the urine sample closest to the >2.0, ≤3.0 h window, after consumption of the meal. Classification performance can be assessed from sample clustering behavior in scores plots representing the first two discriminant functions (DFs) and from quantitative modelling output measures including RF margin values and AUC values.^26^ In both positive (**Figure** 3A) and negative (Figure 3B) ionization modes urine fingerprints showed characteristic differences in the composition of urines collected at different times on one experimental day and the following FMV and fasting urines collected the next morning. The FMV and fasting urines collected on the experimental day overlapped (i.e., were compositionally similar) as were the FMV and fasting urines collected the next morning (with RF margins <0.2 and AUC values <0.8 as shown in Supporting Information 9). Post‐breakfast and post‐lunch samples clustered together with poor classification RF margins and AUC values, but were distinctive from the post‐dinner urine samples, with RF margins >0.2 and AUC values >0.8 (Supporting Information 9). Although the post‐dinner, overnight, and next morning FMV samples showed loose clustering (Figure 3), there are clearly substantial compositional differences, especially in negative mode (Supporting Information 9).

These data suggest that in a real world situation participants would not need to adhere to collecting spot urine samples at strictly pre‐determined times; there would be room for some deviation based on substantial phases (several hours) of the day. These data demonstrated potential for a reduced sampling procedure for both biomarker discovery and for informative habitual dietary exposure measurements in future clinical trials and nutritional status surveys.

### 3.5 Validation of Study Design Performance of Existing Biomarkers Using Spot FMV Samples

The first menu plan (*n* = 15) (Table 2) included instant coffee, wholegrain (sourdough rye bread), high‐sugar food (sweetened breakfast cereal), oily fish (salmon and tuna), a cruciferous vegetable (broccoli), foods for which putative biomarkers have been identified.^15^, ^29^, ^30^, ^31^, ^32^, ^33^, ^34^, ^35^ Using UHPLC‐MS/MS we observed higher concentrations of the known biomarkers for coffee, wholegrain, fish, poultry, and broccoli in FMV urine on the day post consumption of these foods compared with baseline concentrations on a day when the foods were not consumed, or consumed in a low amount (**Table** 3). In contrast there was no significant difference in sucrose concentrations after consumption of sweetened breakfast cereal compared with other menu plans. The means and standard errors for each menu day, with the selected reaction monitoring (SRM) fragments, LOD and LOQ values are shown in Supporting Information 5.

**Table 3:** Concentration in first morning void (FMV) urine samples of proposed biomarkers after consumption of target foods compared to the baseline levels when the food of interest was not consumed, or consumed in a low amount

Food	Proposed biomarker	Concentration in FMV urine the day after consumption [µg mL^−1^, ± st error]	Baseline concentration in FMV urine
Coffee	Feruloylglycine^35^	0.78 ± 0.17**	0.11 ± 0.02
Wholegrain	DHPPA ‐3‐sulfate^31^	1.32 ± 0.16**	0.28 ± 0.02
Broccoli	SFN‐NAC^29^	1.35 ± 0.26**	0.03 ± 0.00
Fish	TMAO^15^, ^32^	41.83 ± 4.79**	9.71 ± 1.02
Poultry and oily fish	L‐Anserine^15^, ^32^	3.97 ± 1.69*	0.30 ± 0.05
Sweetened foods	Sucrose^30^	0.28 ± 0.14	0.54 ± 0.15

DHPPA, 3‐(3,5‐dihydroxyphenyl)‐1‐propanoic acid; TMAO, trimethylamine‐N‐oxide; SFN‐NAC, D,L‐sulforaphane‐N‐acetyl‐L‐cysteine; Paired *t*‐test (*n* = 15) **, *p*‐value <0.01; *, *p*‐value <0.05.

## 4 Discussion

Currently, chemical biomarkers suitable for monitoring dietary exposure are limited to a relatively small number of specific foods and food components.^7^, ^8^, ^9^ Dietary intervention approaches generally used for biomarker discovery usually involve participants consuming a single test food in isolation in a single meal^36^ or repeated foods^37^ or repeated meals with single food components altered.^15^, ^29^ Here we used quantitative information on individual‐level food intake in the UK^11^ and UK healthy eating advice^19^ (Table 1 and Supporting Information 1) to select candidate foods/food groups for incorporation into two complex 3‐day experimental periods in the context of conventional UK eating patterns. Importantly this included information on cooking/preparation methods of commonly consumed foods which we used to test biomarker generalizability by using different formulations/cooking/processing methods for these foods. Analysis of the database (Table 1 and Supporting Information 1) revealed target foods and food groups for incorporation into the menu design to facilitate future biomarker discovery for these food groups (Table 2 and Supporting Information 3).

Individuals in their home‐settings adhered to the menus successfully, with more than 80% of all food items consumed. The lowest acceptability was for instant coffee but it should be noted participants were asked to consume three cups of instant coffee in that experimental menu and that consumption at breakfast and lunchtime (97% and 85% consumption, respectively) was much higher than that for the third cup (80%) consumed in the evening. The overall high compliance (≥80%) for consumption of all food items on the menus provided a robust basis for investigation urine samples by metabolomic approaches.

To be useful for biomarker‐based monitoring of food intake, a urine sampling procedure should be acceptable for participants, require minimal researcher time and deliver samples with high‐quality information content. Since 24‐h urine collections are laborious and unacceptable to some participants,^38^, ^39^ we determined the utility and acceptability of a urine sampling protocol in the home environment based on the collection of spot samples (Figure 1). Participants were asked to consume the test meals at pre‐determined times and to collect spot urine samples within particular time‐windows to investigate which urine samples are most useful for identifying putative biomarkers of recent exposure to target foods. Metabolome fingerprints of urine samples were generated by non‐targeted FIE‐HRMS. Our data suggest that collection of spot FMV and post‐dinner urine samples, rather than overnight or 24 h collections would ensure increased compliance. This would reduce participant burden and would be less intrusive for participants sampling urines in their own home in future by requesting only a spot FMV and/or a post‐dinner sample rather than the collection of 24 h urine. It been has shown previously that the information content of overnight pools and spot fasting urine samples could all adequately discriminate exposure class for several dietary components, and could possibly adequately substitute for 24‐h urines.^14^

The metabolite composition of FMV was generally chemically different from both the overnight and post‐dinner urine collections and varied due to food consumed on the previous menu plan day. The post‐dinner urine represents acute exposure to all foods consumed over the day and the FMV contains these same acute signals and biotransformed derivatives thereof, in addition to representing the activity of the gut microbiome on food ingested the day before. By looking at the micturition timings and success rate of urine sampling from the first 15 participants it was indicated that the most successful urine type collected was FMV and the post dinner spot sample (both at 99% compliance), when compared with fasting and the other post‐prandial urines. We demonstrated that there was an overlap in chemical composition between two pairs of behavioral phase urine types, namely: FMV and fasting, and then the post‐breakfast and post‐lunch sample (Supporting Information 9). This suggests that in a real world situation participants would not need to adhere to collecting spot urine samples at strictly pre‐determined times; there would be room for some deviation based on substantial phases (several hours) of the day. This demonstrated value for a reduced sampling procedure for both biomarker discovery and validation and for informative habitual dietary exposure measurements in future clinical trials and nutritional status surveys.

Our data support the hypothesis that the chemical composition of a spot sample of FMV urine is suitable for identifying biomarkers of commonly consumed foods. Urinary concentrations of reported biomarkers in Table 3 were increased significantly after consumption of the specific food when compared with experimental day 2 and/or 3 when the food was not consumed. Several biomarkers remained significantly increased after a further menu plan for reasons including repeat exposure to foods (i.e., Wholegrain: DHPPA‐sulfate and Poultry and Fish: anserine) and possibly slower biomarker clearance rate (i.e., Coffee: feruloylglycine and Fish: TMAO). There was no significant difference for sucrose^30^ after menu plan 1 compared with other menu plans possibly due to the moderate sugar content of many foods and beverages being consumed on all three days.

By showing that the FMV and post‐dinner urine were the most successful urine types collected by the participants, we propose that in future population health surveys, the request for either replicate FMV and/or post‐dinner urine collections would ensure increased compliance from participants. By collecting spot FMV and post‐dinner urine samples only, rather than overnight or 24‐h collections, we feel it is less intrusive for volunteers and delivers samples with value both for biomarker discovery and validation and for informative dietary exposure measurements. The urine sampling protocols and procedures we designed and implemented could be applied in future large‐scale epidemiological studies and public health surveys, focusing on approaches that were easy to use, acceptable to the general public and which would be of modest cost.

To summarize, we developed a food exposure strategy following a conventional UK eating pattern for biomarker‐lead discovery and validation in free‐living individuals. A urine sampling methodology has been evaluated for free‐living study participants that is non‐intrusive and imposes low burden, and showed that FMV spot urine had good information content in regard to dietary exposure. This study design is expected to have value for both biomarker validation and for informative habitual dietary exposure measurements in epidemiological studies and in population health surveys.

## Conflict of Interest

The authors declare no conflict of interest.

## Supporting information

Supporting Information
